# A Negative (1,3)-β-D-Glucan Result Alone Is Not Sufficient to Rule Out a Diagnosis of *Pneumocystis* Pneumonia in Patients With Hematological Malignancies

**DOI:** 10.3389/fmicb.2021.713265

**Published:** 2021-08-11

**Authors:** Céline Damiani, Baptiste Demey, Cécile Pauc, Yohann Le Govic, Anne Totet

**Affiliations:** ^1^Laboratoire de Parasitologie-Mycologie, Centre de Biologie Humaine, CHU Amiens-Picardie, Amiens, France; ^2^Agents Infectieux, Résistance et Chimiothérapie (AGIR), UR 4294, Université de Picardie Jules Verne, Amiens, France

**Keywords:** *Pneumocystis* pneumonia, biomarker, *Pneumocystis jirovecii*, bronchoalveolar lavage fluid, HIV-negative patients, (1,3)-β-D-glucan, fungal load, hematological malignancies

## Abstract

**Background:** Serum (1,3)-β-D-glucan (BG) testing is increasingly being used in the diagnostic armamentarium for invasive fungal diseases. Given its high sensitivity, some studies suggest that a negative BG result contributes to rule out a diagnosis of *Pneumocystis* pneumonia (PCP). However, recent reports described a suboptimal sensitivity in HIV-negative immunocompromised patients. In this study, we evaluated the performance of BG assay for PCP diagnosis in HIV-negative patients with diverse PCP risk factors. We also assessed the correlation between *Pneumocystis jirovecii* load in pulmonary samples and serum BG levels.

**Methods:** We retrospectively included HIV-negative patients with microscopically proven PCP and for whom a BG result was available. We also enrolled patients colonized by *Pneumocystis* as control group. Colonized patients were matched with PCP patients based on their underlying condition that exposed to PCP. Pulmonary fungal loads were determined by an in-house real-time PCR, and BG levels were measured by using the Fungitell® kit (Associates of Cape Cod, Inc.).

**Results:** Thirty-nine patients were included in each of the two groups. Thirty-four of 39 PCP patients and one of 39 colonized patient had a positive BG test, resulting in a sensitivity of 0.87 (95% CI: 0.73–0.94), a specificity of 0.97 (95% CI: 0.87–0.99), a positive predictive value of 0.97 (95% CI: 0.85–0.99), and a negative predictive value of 0.88 (95% CI: 0.75–0.95) for BG assay. Nonetheless, median BG level differed according to the underlying condition. Among the PCP group, the lowest median level of 211 pg/ml was observed in patients with hematological malignancy (HM) and differed significantly from that observed either in solid organ transplants (3,473 pg/ml) or in patients with autoimmune or inflammatory disorder (3,480 pg/ml). Indeed, the sensitivity of BG assay was estimated at 0.64 (95% CI: 0.35–0.85) in HM patients and was lower than the one observed in the whole PCP group. Furthermore, BG level and fungal burden correlated poorly among all PCP patients.

**Conclusion:** BG is not a reliable biomarker for ruling out PCP in HIV-negative patients with HM. Interpretation of a negative BG result should take into account, but not be limited to, the underlying condition predisposing to PCP.

## Introduction

*Pneumocystis jirovecii* is a transmissible fungus and the causative agent of *Pneumocystis* pneumonia (PCP) in immunocompromised patients. Clinical manifestations include fever, non-productive cough, and dyspnea, which are not specific to PCP. Definite diagnosis traditionally relies on microscopic visualization of asci and/or trophic forms in respiratory samples. Importantly, fungal load varies according to the clinical background, especially the human immunodeficiency virus (HIV) status. Indeed, PCP in HIV-infected patients is mostly associated with high pulmonary *P. jirovecii* burdens, while non-HIV-infected immunocompromised patients usually develop PCP with low fungal burdens, which may result in false-negative microscopic detection (Limper et al., 1989). To overcome this issue, highly sensitive techniques with different cost-sensitivity ratios have been developed (Harris et al., 2011). Nowadays, DNA amplification by polymerase chain reaction (PCR) is widely used. Nevertheless, a positive DNA amplification test is not sufficient alone to distinguish between PCP and colonization (1,3)-β-D-glucan (BG) assays are being used increasingly as adjunctive tools for PCP diagnosis (Esteves et al., 2015; White et al., 2017), but this marker lacks specificity as serum BG can be detected in diverse invasive fungal diseases (IFDs), except for cryptococcosis, mucormycosis, and blastomycosis (Obayashi et al., 1995). Indeed, BG represents a major structural component of the cell walls of most fungi. Thought to be rare or absent in *Pneumocystis* trophic forms, it is synthesized throughout the microorganism life cycle; it is fully formed and abundant in the asci (Dei-Cas et al., 2005). High BG levels were widely reported in patients developing PCP regardless of HIV status (Shimizu et al., 2005; Fujii et al., 2007; Pisculli and Sax, 2008; de Boer et al., 2011; Held et al., 2011; Matsumura et al., 2011; Sax et al., 2011; Damiani et al., 2013; Wood et al., 2013; Dichtl et al., 2018; Hammarström et al., 2019; Sun et al., 2021; Zubkowicz et al., 2021). In their meta-analysis, Karageorgopoulos et al. (2013) mentioned that the sensitivity of BG assay reached 95% for PCP diagnosis. Given this high accuracy, it was proposed that serum BG assay could be used as a screening test, especially when a bronchoalveolar lavage fluid (BALF) retrieval for *P. jirovecii* detection cannot be performed. In this context, it was suggested that a negative BG result could accurately exclude a PCP diagnosis (Held et al., 2011; Onishi et al., 2012; Alanio et al., 2016; Lahmer et al., 2017; Morjaria et al., 2019; Ideguchi et al., 2020; Szvalb et al., 2020). In contrast, other reports describe a suboptimal sensitivity of this assay, especially in HIV-negative patients (Nakamura et al., 2009; Li et al., 2015; Engsbro et al., 2019; Kato et al., 2019; Del Corpo et al., 2020; Mercier et al., 2020; Rogina and Skvarc, 2020). These discrepant results regarding the usefulness of serum BG assay in HIV-negative PCP patients could be related to the underlying condition that exposes to PCP risk. The most vulnerable patients are those suffering from hematological malignancies (HM) or solid cancers from systemic autoimmune and inflammatory disorder (SAIID) and also patients who have undergone solid organ transplantation (SOT) or hematopoietic stem cell transplantation (Roux et al., 2014; Dunbar et al., 2020; Fishman, 2020). Of note, a lower sensitivity of BG assays was reported for other IFDs in patients with HM (Lamoth et al., 2012, 2021; Giacobbe et al., 2017). Taken together, these data question the accuracy of serum BG to rule out PCP diagnosis in non-HIV-infected populations, especially in patients with HM.

Therefore, the aim of this study was to assess the performance of BG assay for PCP diagnosis in a series of HIV-negative patients. We also assessed the correlation between pulmonary loads of *P. jirovecii* and serum BG levels. For these purposes, we conducted a 10-year single-center retrospective study focusing on non-HIV-infected hematology and non-hematology patients.

## Materials and Methods

This retrospective study was performed at the University Hospital of Amiens, Picardy, France, between May 2011 and January 2021.

### Inclusion Criteria

All non-HIV-infected patients aged ≥18 years with proven PCP for whom one BG result was available were retrospectively enrolled. Proven PCP was defined as a positive microscopic detection of *P. jirovecii* in BALF sample.

### Control Group

In order to perform statistical analyses, a control group of patients colonized by *P. jirovecii* was established. Colonization is defined by a positive PCR assay and a negative microscopic detection of the fungus in patients for whom a clinical improvement was obtained in the absence of *Pneumocystis* specific treatment. Colonized patients were matched with PCP patients according to their underlying condition.

### *Pneumocystis* Detection

*Pneumocystis jirovecii* was detected in BALF samples by microscopic examination with methanol-Giemsa staining. This staining was combined with an immunofluorescence assay (Monofluokit *Pneumocystis*; Bio-Rad, Marnes-La-Coquette, France) during the first 7 years of the study and thereafter by using a stilbene-derived fluorescent dye (Uvibio®; LDBIO Diagnostics, Lyon, France).

*Pneumocystis jirovecii* DNA was also systematically detected by PCR. DNAs were directly extracted from 1 ml BALF sample and eluted in 100 μl buffer using the automated NucliSENS® easyMAG® (bioMérieux, Marcy l’Etoile, France) platform. An in-house real-time PCR assay targeting the mitochondrial large subunit rRNA gene was then performed as previously described (Totet et al., 2003).

### BG Assays

BG levels were determined by using the Fungitell® kit (Associates of Cape Cod, Inc., Cape Cod, MA) according to the manufacturer’s instructions. The BG concentrations in samples were calculated automatically by using a calibration curve established with standard solutions ranging from 31 to 500 pg/ml. A cut-off BG level of ≥80 pg/ml was considered as a positive test result as defined by the manufacturer. Assays were performed in duplicate. Samples with levels higher than 500 pg/ml were 10-fold diluted and reassayed. After dilution, samples with levels higher than 5,000 pg/ml were not diluted again and the value 5,000 pg/ml was considered for statistical analysis. BG levels below 31 pg/ml (lower quantification limit) were systematically set at 31.

### Data Collection

We reviewed the medical records of patients enrolled in the study and the following demographic and clinical variables were collected: age, sex, and underlying condition that exposed to PCP. We also checked for PCP prophylaxis, intravenous immunoglobulin (IVIG) therapy, or methotrexate use at the time of presentation. We compiled laboratory findings including cycle threshold (Ct) values of qPCR assays as well as results of serum BG testing (expressed in pg/ml) obtained within 14 days before or after BALF sampling. When available, the results of galactomannan and mannan antigen testing as well as those of fungal and bacterial cultures were also collected.

### Statistical Analysis

Statistical analysis and graphic representations were performed using GraphPad Prism version 5.00 for Windows (GraphPad Software, San Diego, CA, United States). Chi-squared test and Fisher’s exact test were used to compare categorical variables. Numerical data with non-Gaussian distributions were reported as medians (range, interquartile range: IQR) and were analyzed with Mann–Whitney test. Numerical data with Gaussian distributions were reported as means (standard deviation: SD) and were analyzed with Student’s test. Kruskal-Wallis test was used for three-group comparisons. If a significant result was observed, Holm–Bonferroni method was then applied for pairwise group comparisons. Receiver operating characteristic (ROC) curves were constructed based on BG results. The sensitivity, specificity, positive predictive value (PPV), and negative predictive value (NPV) were computed with 95% confidence interval (95% CI). Statistical relationship between pulmonary load of *P. jirovecii* (expressed in Ct) and serum BG value (expressed in pg/ml) was assessed using Spearman’s rank correlation. Statistical significance was defined as a value of *p* ≤ 0.05.

### Ethics Statement

In line with the French legislation concerning non-interventional retrospective studies, patient informed consent was waived. However, according to the reference method MR-004 published by the French Data Protection Authority (CNIL), the study was registered by our Institutional Review Board under number PI2021_843_0078.

## Results

### Patients

Thirty-nine patients with proven PCP met the inclusion criteria, and descriptive characteristics are summarized in Table 1. The median age was 65 years (range 26–78, IQR 11) and the sex ratio was 2. None of the 39 patients received anti-*Pneumocystis* drugs before BALF sampling. Two patients (P_8_ and P_9_) developed an invasive candidiasis concurrently with the PCP. No others presented any clinical or laboratory signs of IFD other than PCP. Three patients had concurrent gram-negative sepsis (P_4_, P_9_, and P_12_). One (P_8_) and six (P_1_, P_4_, P_10_, P_12_, P_26_, and P_34_) patients received IVIG therapy or methotrexate administration at the time of PCP diagnosis, respectively. All patients received anti-*Pneumocystis* treatment within the 24 h following BALF sampling. Number of days between serum and BALF sampling ranged from 0 to 10 days (median, 1 day). Clinical improvement was observed in 30 of 39 patients (77%). The nine remaining patients died within 30 days after PCP diagnosis. There were five SAIID patients (P_9_, P_15_, P_26_, P_34_, and P_38_), three HM patients (P_10_, P_12_, and P_28_), and one patient with solid cancer (P_26_).

**Table 1:** Characteristics of the 39 patients developing microscopically proven *Pneumocystis* pneumonia.

Patient no.	Age (years)	Gender	Underlying condition	30-day outcome	PCR Ct value in BALF sample	Serum BG level (pg/ml)	Time between serum and BALF sampling (days)
Patients with systemic autoimmune or inflammatory disorder							
P_1_^§^	59	F	Sarcoidosis	Survived	19	3,480	4
P_9_^*^^,^^#^	69	M	Psoriatic arthritis	Died	27	4,550	1
P_15_	65	M	Drug-induced interstitial lung disease	Died	26	193	0
P_26_^§^	78	M	Rheumatoid arthritis	Died	30	3,885	3
P_32_	54	M	Crohn’s disease	Survived	26	2,794	2
P_34_^§^	63	M	Sarcoidosis	Died	19	2,979	2
P_37_	69	F	Hashimoto’s thyroiditis	Survived	22	3,510	0
P_38_	51	F	Focal segmental glomerulosclerosis	Died	20	>5,000	1
P_39_	69	M	Idiopathic thrombocytopenic purpura	Survived	19.5	610	1
Patients with solid organ transplant							
P_2_	77	M	Kidney transplant	Survived	21	3,952	0
P_3_	67	F	Kidney transplant	Survived	26.5	1,290	0
P_6_	65	F	Kidney transplant	Survived	18	1,170	2
P_7_	69	F	Kidney transplant	Survived	15	3,400	0
P_8_^*^^,^^$^	68	M	Kidney transplant	Survived	16	3,507	3
P_13_	58	M	Kidney transplant	Survived	26	4,075	0
P_19_	67	M	Liver transplant	Survived	18.5	3,410	2
P_20_	67	M	Kidney transplant	Survived	21	>5,000	1
P_21_	36	F	Kidney transplant	Survived	19	3,439	1
P_22_	58	M	Kidney transplant	Survived	23	4,094	0
P_24_	55	F	Kidney transplant	Survived	18	>5,000	2
P_25_	62	M	Kidney transplant	Survived	20	2,738	4
P_27_	33	M	Kidney transplant	Survived	25	2,330	1
P_29_	61	M	Kidney transplant	Survived	21	>5,000	2
P_33_	76	F	Kidney transplant	Survived	22	2057	5
P_35_	69	M	Heart transplant	Survived	24	>5,000	0
Patients with hematological malignancy							
P_4_^#^^,^^§^	58	F	T-cell large granular lymphocyte leukemia	Survived	26.5	2,771	1
P_5_	64	F	Angioimmunoblastic T-cell lymphoma	Survived	30	1900	2
P_10_^§^	70	M	Diffuse large B-cell lymphoma	Died	28	269	1
P_11_	67	M	Diffuse large B-cell lymphoma	Survived	28.5	211	1
P_12_^#^^,^^§^	56	M	Burkitt lymphoma	Died	19	1,478	0
P_14_	58	M	Hodgkin lymphoma	Survived	27.5	117	1
P_16_	57	M	Mantle cell lymphoma	Survived	26	<31	1
P_17_	26	M	Hodgkin lymphoma	Survived	26	<31	1
P_28_	67	M	Hodgkin lymphoma	Died	26	77	10
P_30_	44	F	Chronic lymphocytic leukemia	Survived	26.5	68	4
P_31_	78	F	Mixed-phenotype acute leukemia	Survived	34	1,557	0
Patients with solid cancer							
P_18_	62	M	Kidney cancer	Survived	25	40	1
P_23_	75	M	Squamous cell lung carcinoma	Survived	23	395	0
P_36_	73	M	Lung adenocarcinoma	Died	18.5	2,890	0

*BALF, bronchoalveolar lavage fluid; BG, (1,3)-β-D-glucan; Ct, cycle threshold; F, Female; M, Male*.

* *Patients with concurrent invasive candidiasis*.

# *Patients with concurrent gram-negative sepsis*.

$ *Patients receiving intravenous immunoglobulin therapy*.

§ *Patients receiving methotrexate administration*.

Thirty-nine patients colonized by *P. jirovecii* were also enrolled. Characteristics are summarized in Table 2. The median age was 67 years (range 38–84, IQR 11) and the sex ratio was 1.44. No patient developed IFD. Three patients had gram-negative sepsis concomitant with PCP (C_4_, C_31_, and C_33_) and two patients were treated with methotrexate at the time of BALF sampling (C_5_ and C_14_). Serum samples were collected over an interval ranging from 13 days before to 14 days after BALF retrieval (median, 0 day). No patient received anti-*Pneumocystis* treatment and clinical improvement was observed in 35 of 39 colonized patients (90%). The four remaining patients died within 30 days after *P. jirovecii* detection by PCR but the fungus was not involved in the death; SAIID patient C_21_ died from sepsis shock, SOT patient C_26_ died from disseminated intravascular coagulation and associated multi-organ failure, and HM patients C_3_ and C_22_ died from progressive myeloma and influenza A induced acute respiratory distress syndrome, respectively.

**Table 2:** Characteristics of the 39 patients colonized by *Pneumocystis*.

Patient no.	Age (years)	Gender	Underlying condition	30-day outcome	PCR Ct value in BALF sample	Serum BG level (pg/ml)	Time between serum and BALF sampling (days)
Patients with systemic autoimmune or inflammatory disease							
C_1_	75	F	Rheumatoid arthritis	Survived	36	57	2
C_4_^#^	71	M	Granulomatosis with polyangiitis	Survived	32.5	<31	−2
C_5_^§^	72	M	Pneumoconiosis	Survived	32.5	<31	0
C_11_	80	F	Systemic lupus erythematosus	Survived	36.5	76	7
C_14_^§^	54	F	Dermatomyositis	Survived	35.5	40	−1
C_18_	65	M	Rheumatoid arthritis	Survived	35.5	75	−1
C_19_	63	F	Granulomatosis with polyangiitis	Survived	37	<31	0
C_21_	72	M	Rheumatic fever	Died	34.5	<31	0
C_31_^#^	51	F	Multiple sclerosis	Survived	35	<31	9
Patients with solid organ transplant							
C_2_	54	M	Kidney transplant	Survived	37	<31	14
C_7_	63	M	Kidney transplant	Survived	37.5	67	8
C_9_	67	M	Kidney transplant	Survived	35	36	14
C_10_	73	M	Kidney transplant	Survived	38	<31	−13
C_13_	78	M	Kidney transplant	Survived	36.5	<31	2
C_15_	48	F	Kidney transplant	Survived	38	<31	0
C_16_	72	F	Kidney transplant	Survived	38	34	4
C_17_	71	M	Kidney transplant	Survived	38	<31	−2
C_20_	62	F	Kidney transplant	Survived	30	<31	0
C_23_	68	F	Kidney transplant	Survived	31.5	44	0
C_26_	80	M	Kidney transplant	Died	34	51	−1
C_28_	62	M	Kidney transplant	Survived	34	<31	0
C_29_	69	F	Kidney transplant	Survived	33	<31	8
C_36_	77	M	Kidney transplant	Survived	32	<31	11
C_37_	30	M	Kidney transplant	Survived	37	<31	−5
C_39_	66	M	Kidney transplant	Survived	37	<31	6
Patients with hematological malignancy							
C_3_	84	M	Follicular lymphoma	Died	36.5	54	0
C_8_	50	F	Acute myeloid leukemia	Survived	38	<31	15
C_22_	77	F	Multiple myeloma	Died	38	<31	−1
C_24_	63	M	Multiple myeloma	Survived	37.5	<31	14
C_25_	80	F	Chronic lymphocytic leukemia	Survived	36.5	69	0
C_27_^#^	38	M	Mixed-phenotype acute leukemia	Survived	35	<31	0
C_30_	55	F	Acute myeloid leukemia	Survived	36.5	<31	−1
C_32_^#^	54	M	Diffuse large B-cell lymphoma	Survived	37.5	<31	−9
C_33_	66	M	Hodgkin lymphoma	Survived	34.5	31	−1
C_34_	68	M	Diffuse large B-cell lymphoma	Survived	38	49	10
C_35_	67	M	Mantle cell lymphoma	Survived	32	57	0
Patients with solid cancer							
C_6_	64	F	Lung adenocarcinoma	Survived	37.5	119	−3
C_12_	67	F	Breast cancer	Survived	35.5	66	0
C_38_	74	M	Lung adenocarcinoma	Survived	32	69	7

*BALF, bronchoalveolar lavage fluid; BG, (1,3)-β-D-glucan; Ct, cycle threshold; F, Female; M, Male*.

# *Patients with concurrent gram-negative sepsis*.

§ *Patients receiving methotrexate administration*.

There was no significant difference between the PCP group and the colonization group with respect to age (*p* = 0.14, Mann–Whitney test) or gender (*p* = 0.48, Chi-squared test; Table 3).

**Table 3:** Statistical analyses of categorical and numerical variables in patients with *Pneumocystis* pneumonia and patients colonized by *Pneumocystis*.

Clinical presentation	PCP (*n* = 39)	Colonization (*n* = 39)	*p*
Median age, years (range; IQR)	65 (26–78; 11)	67 (38–84; 11)	0.14^#^
Male gender, *n* (%)	26 (66.7)	23 (59)	0.48^$^
Positive BG result, *n* (%)	34	1	<10^–14^^*^^,^^§^
Serum BG level, pg/ml, median (range; IQR)	2,771 (31–5,000; 3,490)	31 (31–119; 23)	<10^–3^^*^^,^^#^
PCR Ct value in BALF samples, median (range; IQR)	23 (15–34; 8)	36.5 (30–38; 32)	<10^–3^^*^^,^^#^

*BALF, bronchoalveolar lavage fluid; BG (1,3)-β-D-glucan; Ct, cycle threshold; IQR, interquartile range; PCP, Pneumocystis pneumonia*.

* *Statistically significant*.

# *Mann–Whitney test*.

$ *Chi-squared test*.

§ *Fisher’s exact test*.

The 39 PCP patients were subsequently assigned to four different subgroups, depending on their underlying condition; 11 (28.2%) and three (7.7%) patients presented with HM and solid cancer, respectively. Sixteen patients (41%) were SOT recipients, and the nine (23.7%) remaining patients received high-dose corticosteroids or immunosuppressive agents for SIIAD. Because of the low sample size, patients with solid cancer were not considered for further statistical analyses.

The median age in the HM subgroup, the SOT subgroup and the SAIID subgroup was 58 (range 26–78, IQR 11), 65 (range 33–77, IQR 10.5), and 65 (range 51–78, IQR 12.5) years, respectively (Table 4). In the same subgroup sequence, the sex ratio was 1.75, 1.67, and 2, respectively. Neither age nor sex differed significantly between the three subgroups (*p* = 0.059 and *p*=0.39, respectively, Kruskal-Wallis test).

**Table 4:** Statistical analyses of variables in patients with *Pneumocystis* pneumonia, according to the underlying condition.

Underlying condition	HM (*n* = 11)	SOT (*n* = 16)	SAIID (*n* = 9)	*p* ^#^
Median age, years (range; IQR)	58 (26–78; 11)	65 (33–77; 10.5)	65 (51–78; 12.5)	0.059
Male gender, *n* (%)	7 (63.6)	10 (62.5)	6 (66.7)	0.39
Serum BG level, pg/ml, median (range; IQR)	211 (31–2,771; 1,489)	3,473 (1170–5,000; 2013)	3,480 (610–5,000; 2,515)	<10–4^#^^,^^$^
PCR Ct value in BALF samples, mean ± SD (range)	27.09 ± 3.59 (19–34)	20.87 ± 3.42 (15–26.5)	23.17 ± 4.14 (19.5–30)	0.0006^#^^,^^$^

*BALF, bronchoalveolar lavage fluid; BG, (1,3)-β-D-glucan; Ct, cycle threshold; HM, hematological malignancy; IQR, interquartile range; PCP, Pneumocystis pneumonia; SD, standard deviation; SAIID, systemic autoimmune or inflammatory disorder; SOT, solid organ transplant*.

# *3-group comparison by Kruskal-Wallis test*.

$ *Statistically significant; pairwise comparison by Bonferroni’s test was subsequently performed*.

Since colonized patients were matched with PCP patients by the underlying condition, the same subgroups were designed. The mean age was 63.8 ± 12.7 (range 38–84), 66.9 ± 8.6 (range 48–80), and 67 ± 9.6 years (range 51–80) and the sex ratio was 1.75, 2.2, and 0.8 in the HM subgroup, the SOT subgroup and the SAIID subgroup, respectively (Table 5). Again, there was no significant difference between the subgroups regarding age or gender (*p* = 0.73 and *p* = 0.45, respectively, Kruskal-Wallis test).

**Table 5:** Statistical analyses of variables in patients colonized by *Pneumocystis*, according to the underlying condition.

Underlying condition	HM (*n* = 11)	SOT (*n* = 16)	SAIID (*n* = 9)	*p* ^#^
Mean age ± SD, years (range)	63.8 ± 12.7 (38–84)	66.9 ± 8.6 (48–80)	67 ± 9.6 (51–80)	0.73
Male gender, *n* (%)	7 (63.6)	11 (68.7)	4 (44.5)	0.45
Serum BG level, pg/ml, median (range; IQR)	31 (31–69; 23)	31 (31–67; 4)	31 (31–76; 35)	0.31
PCR Ct value in BALF samples, mean ± SD (range)	36.37 ± 1.88 (32–38)	35.41 ± 2.66 (30–38)	35 ± 1.6 (32.5–37)	0.24

*BALF, bronchoalveolar lavage fluid; BG (1,3)-β-D-glucan; Ct, cycle threshold; HM, hematological malignancy; IQR, interquartile range; SD, standard deviation; SAIID, systemic autoimmune or inflammatory disorder; SOT, solid organ transplantation*.

# *3-group comparison by Kruskal-Wallis test*.

### Performance of BG Assay for PCP Diagnosis

**Figure 1:**
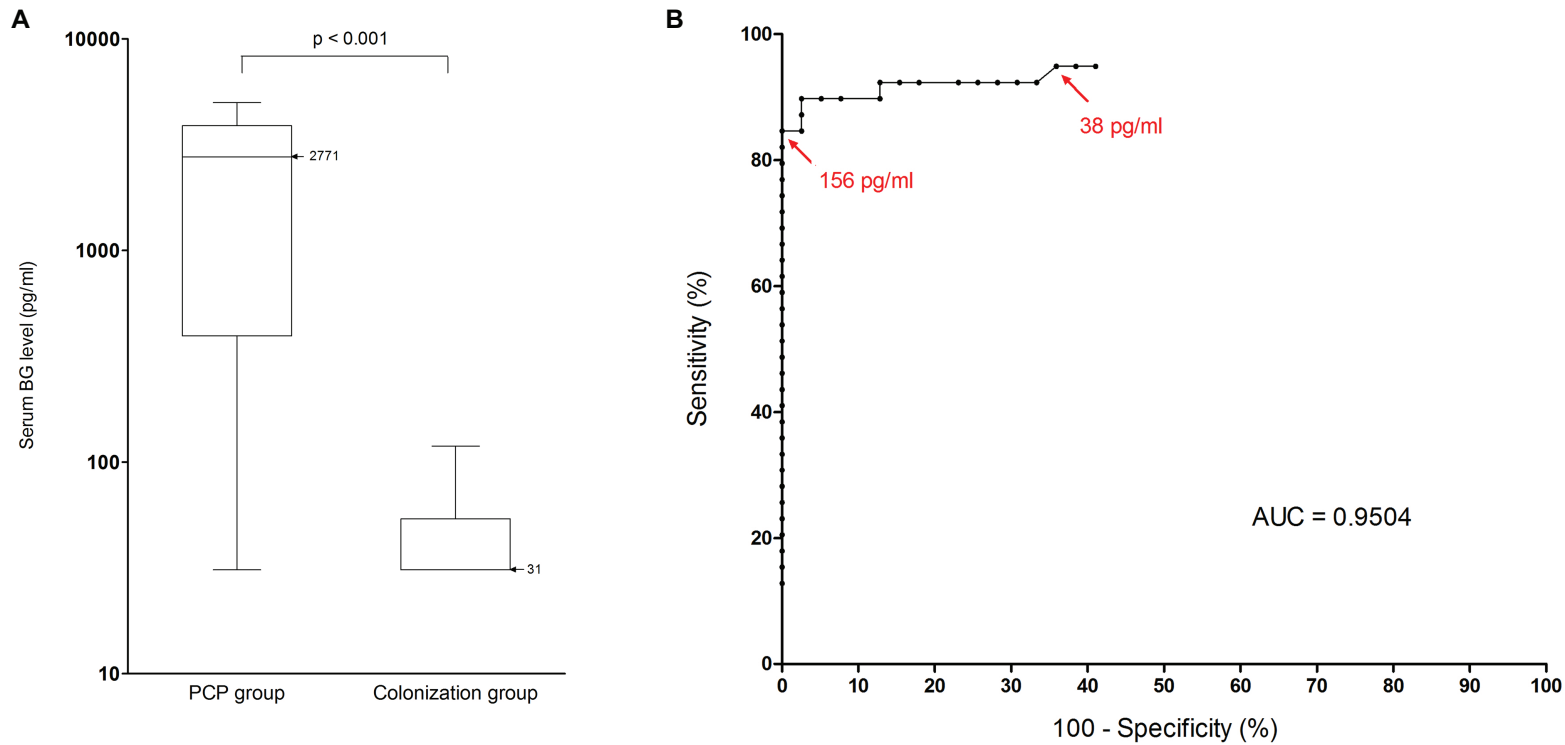
Performance of BG assay for *Pneumocystis* pneumonia diagnosis in HIV-negative population. **(A)** Serum BG levels (pg/ml) in patients with *Pneumocystis* pneumonia and in colonized patients. Black horizontal bars, median values. The median value for PCP patients was 2,771 pg/ml (range, 31–5,000; interquartile range, 3,490 pg/ml). The median value for colonized patients was 31 pg/ml (range, 31–119; interquartile range, 23 pg/ml). Serum BG levels were significantly lower in the colonization group than in the PCP group (Mann–Whitney test, *p* < 0.001). **(B)** Receiver operating characteristic curve for serum BG assay performance, using microscopic detection of *Pneumocystis jirovecii* as the reference method. Red arrows represent calculated thresholds for maximal specificity (156 pg/ml) and maximal sensitivity (38 pg/ml). AUC, area under the curve; BG, (1,3)-β-D-glucan; and PCP, *Pneumocystis* pneumonia.

Thirty-four of 39 PCP patients and one of 39 colonized patients had a positive BG result, which is significantly different from each other (*p* < 10^−14^, Fisher’s exact test, Table 3). Furthermore, the median BG levels in the PCP group and the colonization group were 2,771 pg/ml (range 31–5,000, IQR 3490) and 31 pg/ml (range 31–119, IQR 23), respectively. Values were significantly lower in the colonization group (*p* < 10^−3^, Mann–Whitney test, Figure 1A). At the manufacturer’s positive threshold of 80 pg/ml, sensitivity, specificity, PPV, and NPV for PCP diagnosis were calculated at 0.87 (95% CI: 0.73–0.94), 0.97 (95% CI: 0.87–0.99), 0.97 (95% CI: 0.85–0.99), and 0.88 (95% CI: 0.75–0.95), respectively (Table 6). By ROC analysis, the optimal threshold to distinguish between PCP and colonization was estimated at the value 156 pg/ml (Figure 1B). Using this cutoff, the specificity reached the maximal value 1 (95% CI: 0.9–1) with a sensitivity of 0.85 (95% CI: 0.69–0.94). Conversely, 100% sensitivity could not be obtained. The highest sensitivity was 0.95 (95% CI: 0.83–0.99) with a cutoff value of 38 pg/ml. It was associated with a specificity of 0.64 (95% CI: 0.47–0.79).

**Table 6:** Performance of (1,3)-β-D-glucan assay for *Pneumocystis* pneumonia diagnosis in overall non-HIV-infected immunocompromised population and in population of patients with hematological malignancy.

	Overall population	HM-population
Sensitivity (95% CI)	87% (73–94)	64% (35–85)
Specificity (95% CI)	97% (87–99)	100% (74–100)
Positive predictive value (95% CI)	97% (85–99)	100% (64–100)
Negative predictive value (95% CI)	88% (75–95)	73% (48–89)

*Performance was determined using the manufacturer’s threshold (80 pg/ml). CI, confidence interval; HM, hematological malignancy*.

**Figure 2:**
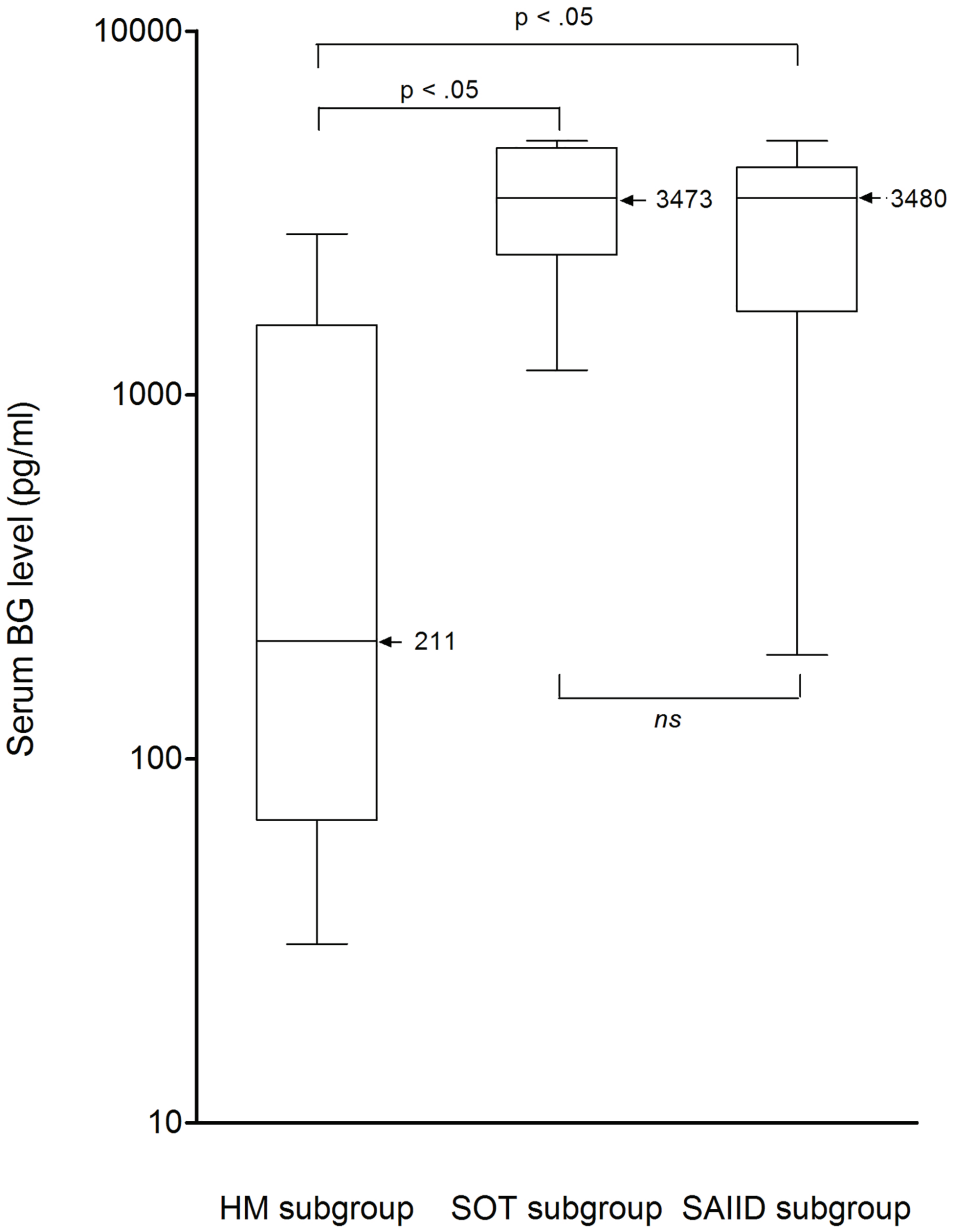
Serum BG levels (pg/ml) in patients with *Pneumocystis* pneumonia from HM, SOT, and SAIID subgroups. Black horizontal bars, median values. The median value for HM patients was 211 pg/ml (range, 31–2,771; interquartile range, 1,489 pg/ml). The median value for SOT patients was 3,473 pg/ml (range, 1,170–5,000; interquartile range, 2,013 pg/ml). The median value for SAIID patients was 3,480 pg/ml (range, 610–5,000; interquartile range, 2,515 pg/ml). Median BG level was significantly lower in the HM subgroup than in the SOT and SAIID subgroups (Bonferroni’s test, *p* < 0.05). No significant difference was observed between SOT patients and SAIID patients. BG, (1,3)-β-D-glucan; HM, hematological malignancy; ns, non-significant; SAIID, systemic autoimmune and inflammatory disorder; and SOT, solid organ transplant.

**Figure 3:**
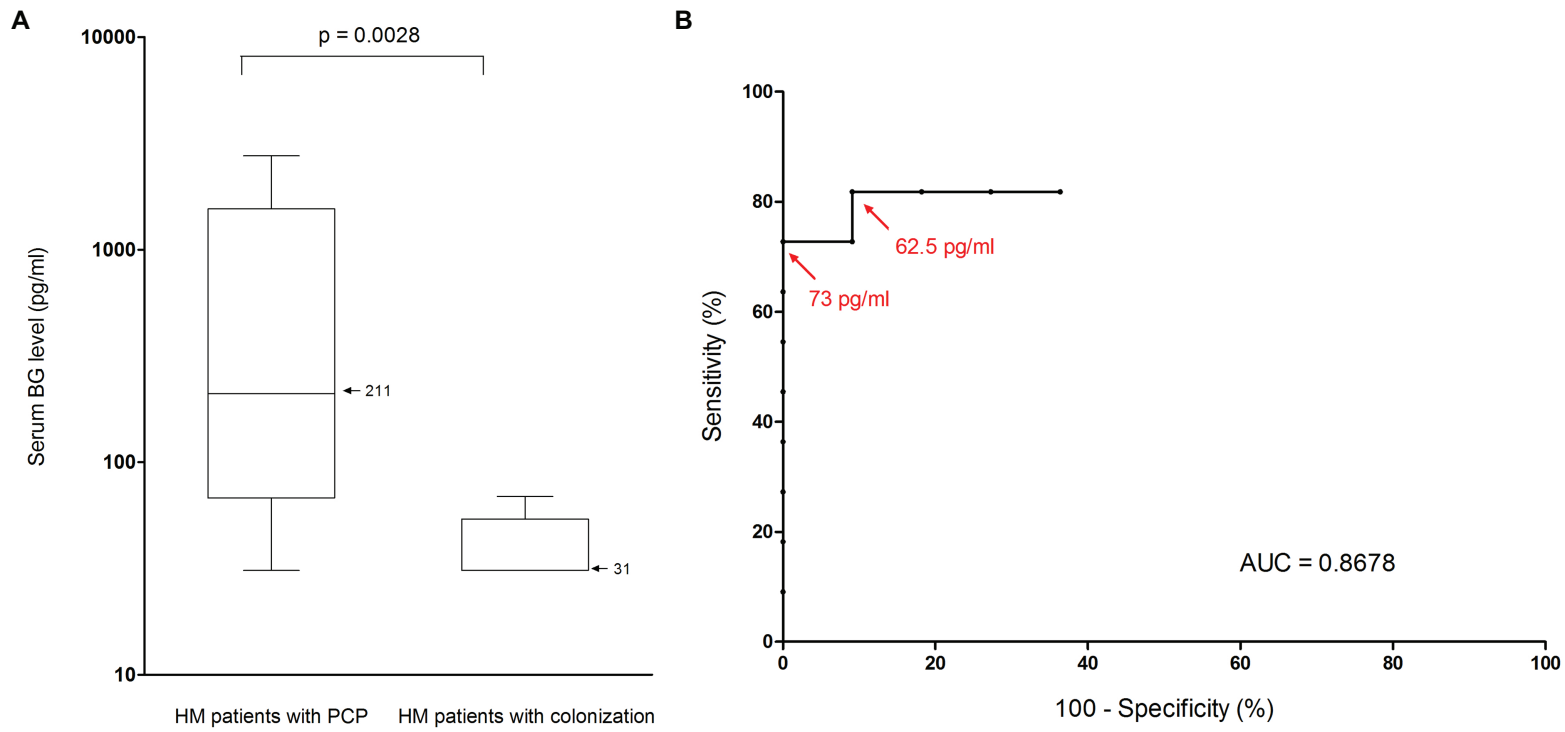
Performance of BG assay for *Pneumocystis* pneumonia diagnosis in patients suffering from hematological malignancy. **(A)** Serum BG levels (pg/ml) in HM patients with *Pneumocystis* pneupneumonia and in HM patients colonized by *Pneumocystis*. Black horizontal bars, median values. The median value for HM patients with PCP was 211 pg/ml (range, 31–2,271; interquartile range, 1,489 pg/ml). The median value for HM patients with colonization was 31 pg/ml (range, 31–69; interquartile range, 23 pg/ml). Serum BG levels were significantly lower in the colonization group than in the PCP group (Mann–Whitney test, *p* = 0.0028). **(B)** Receiver operating characteristic curve for serum BG assay performance in HM patients, using microscopic detection of *P. jirovecii* as the reference method. Red arrows represent calculated thresholds for maximal specificity (73 pg/ml) and maximal sensitivity (62.5 pg/ml). AUC, area under the curve; BG, (1,3)-β-D-glucan; HM, hematological malignancy; and PCP, *Pneumocystis* pneumonia.

Nonetheless, among PCP patients, median BG level differed according to the underlying condition (*p* < 10^−4^, Kruskal-Wallis test, Table 4). Indeed, median level was significantly lower in the HM subgroup than in the SOT and SAIID subgroups (211 vs. 3,473 and 3,480 pg/ml respectively, *p* < 0.05, Bonferroni’s test, Figure 2). The difference remained significant after excluding the two patients presenting with a concurrent invasive candidiasis (SOT patient P_8_ and SAIID patient P_9_). By contrast, there was no significant difference between SOT patients and SAIID patients. Because of significant lower level in HM patients, the performance of BG assay was further especially assessed in this patient subgroup. As in overall patient population, median level was significantly higher in the HM-PCP group than in the HM-colonization group (211 vs. 31 pg/ml, *p* = 0.0028, Mann–Whitney test, Figure 3A). At 80 pg/ml threshold, sensitivity, specificity, PPV, and NPV for PCP diagnosis were calculated at 0.64 (95% CI: 0.35–0.85), 1 (95% CI: 0.74–1), 1 (95% CI: 0.64–1), and 0.73 (95% CI: 0.48–0.89), respectively (Table 6). The ROC curve was used to determine the optimal cutoff value for discriminating PCP from colonization in HM subgroup. This was calculated at the value 73 pg/ml resulting in an improved specificity of 1 (95% IC: 0.9–1) with a related sensitivity of 0.73 (95% IC: 0.39–0.94; Figure 3B). Once more, 100% sensitivity could not be reached and the maximal value was 0.82 (95% CI: 0.48–0.98) with a threshold at 62.5 pg/ml. The corresponding specificity was 0.91 (95% CI: 0.59–0.99).

In other respects, among colonized patients, median BG level did not differ significantly between the three previously defined subgroups (*p* = 0.31, Kruskal-Wallis test, Table 5; Supplementary Figure S1).

### Ct Values in BALF Samples

**Figure 4:**
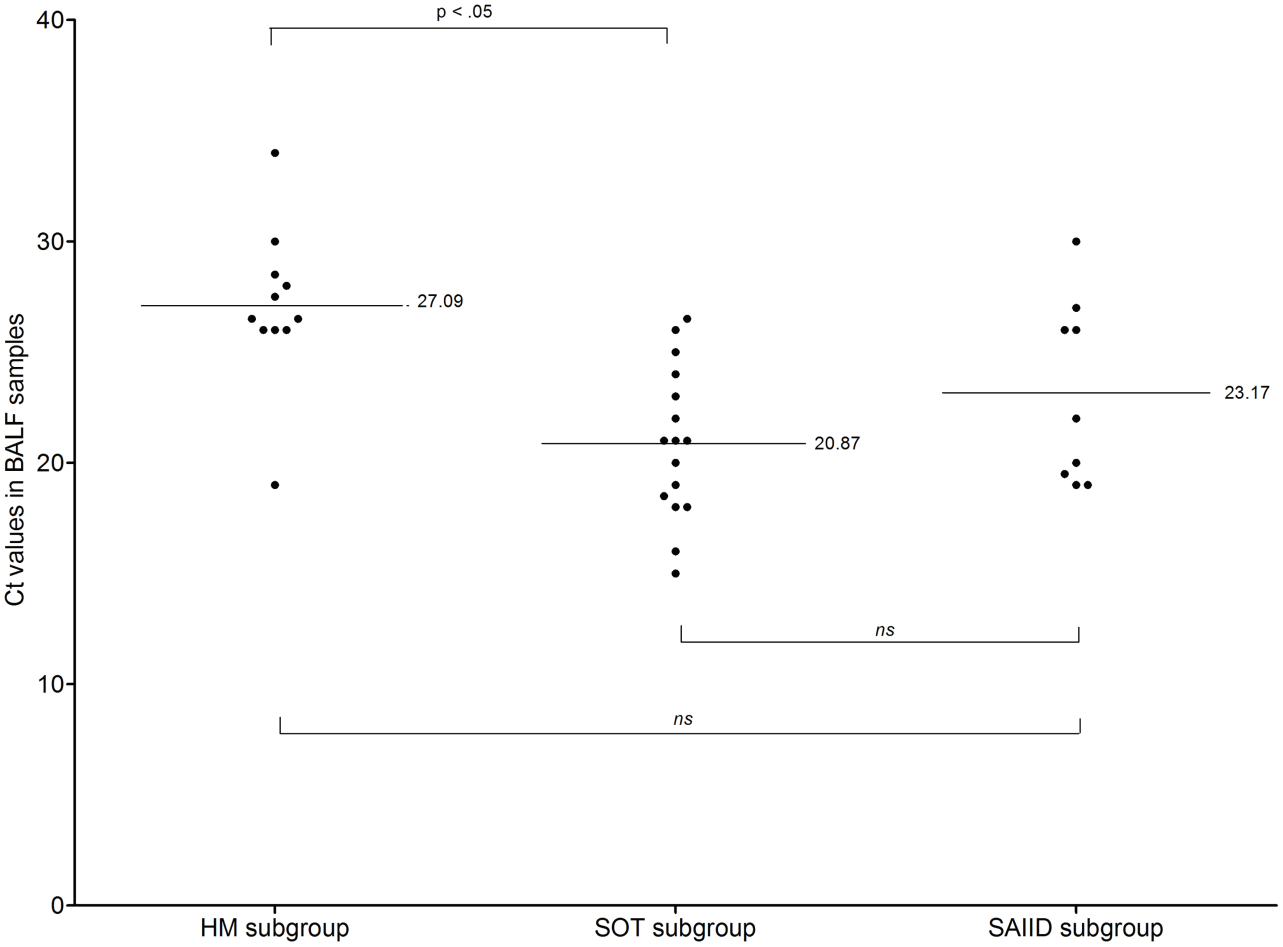
Ct values in patients with *Pneumocystis* pneumonia from HM, SOT, and SAIID subgroups. Black horizontal bars, mean values. The mean value for HM patients was 27.09 ± 3.59 (range, 19–34). The mean value for SOT patients was 20.87 ± 3.42 (range, 15–26.5). The mean value for SAIID patients was 23.17 ± 4.14 (range, 19.5–30). Mean Ct value was significantly lower in SOT patients than in HM patients (Bonferroni’s test, *p* < 0.05). No significant difference was observed between SOT patients and SAIID patients or between HM patients and SAIID patients. BALF, Bronchoalveolar lavage fluid; Ct, cycle threshold; HM, hematological malignancy; ns, non-significant; SAIID, systemic autoimmune and inflammatory disorder; and SOT, solid organ transplant.

Median Ct value was significantly lower in the PCP group than in the colonization group (23 vs. 36.5, *p* < 10^−3^, Mann–Whitney test, Table 3). As above described for BG concentration, mean Ct value differed among PCP patients, depending on the underlying condition (*p* = 0.0006, Kruskal-Wallis test, Table 4); this was significantly lower in SOT patients than in HM patients (20.87 vs. 27.09, *p* < 0.05, Bonferroni’s test, Table 4; Figure 4) while mean value in SAIID patients did not differ from that in either SOT or HM patients. In colonization group, no difference was observed between the three subgroups (*p* = 0.24, Kruskal-Wallis test, Table 5; Supplementary Figure S2).

### Correlation Between PCR Ct Values in BALF Samples and Serum BG Levels

A correlation analysis between Ct values and BG levels was performed in all 39 PCP patients, regardless of their underlying condition (Supplementary Figure S3).

A low negative correlation was found before (Spearman’s correlation coefficient *ρ* = −0.37, *p* = 0.018) and after (*ρ* = −0.43, *p* = 0.0083), excluding patients presenting with a concurrent invasive candidiasis.

We further examined whether the time interval between BALF and serum sampling weakened the correlation between Ct values and BG levels. Spearman’s test was then performed after excluding the 12 patients for whom a serum sample was collected on the same day as BALF sample. Again, we found a low negative correlation and the coefficient remained almost unchanged (*ρ* = −0.4, *p* = 0.03).

## Discussion

*Pneumocystis jirovecii* remains a major cause of life-threatening pneumonia in immunocompromised hosts. Nowadays, diagnostic confirmation still relies on microscopic visualization of the organisms directly from BALF samples, while quantitative PCR assays cannot always distinguish colonization from pneumonia. Given the invasiveness of collecting BALF specimens and the expertise needed to interpret *P. jirovecii* test results, there is growing interest in measuring serum biomarkers such as BG to aid in the diagnosis of PCP. However, the sensitivity of BG testing seems variable according to the underlying disease, especially among non-HIV-infected patients.

Microscopic detection of *P. jirovecii* was our main inclusion criteria and all PCP patients enrolled in this study developed clinical and radiological signs of pneumonia as recently defined by the Organization for Research and Treatment of Cancer and the Mycoses Study Group Education and Research Consortium (EORTC/MSGERC; Lagrou et al., 2021). Therefore, they all presented with a proven PCP. To the best of our knowledge, this is the first study focusing on BG assay performance that did not consider probable PCP cases, i.e., PCP related to PCR based-diagnosis. Indeed, a positive qPCR result associated with a negative microscopic detection of *P. jirovecii* can be consistent with either PCP or pulmonary colonization. In this latter case, BG levels are usually low (Damiani et al., 2011, 2013). Our stringent criteria – allowing inclusion of only proven PCP – ensured that negative BG results could really be observed in the course of PCP and were not related to colonization misclassified as PCP.

At the manufacturer’s threshold, we showed in non-HIV-infected patients that BG assay yielded an overall sensitivity of 87% and a corresponding NPV of 88% for PCP diagnosis, regardless of the underlying condition. These results are in accordance with previous reports that have emphasized suboptimal performance of BG assays in HIV-negative patient populations, with a pooled sensitivity of 83–89% (Li et al., 2015; Engsbro et al., 2019). Moreover, a recent meta-analysis indicated that BG testing cannot reliably rule out PCP in high-risk patients (Del Corpo et al., 2020). Indeed, the authors showed that a negative BG result was not associated with a low post-test probability of PCP when the pre-test probability was moderate to high, which precludes its use as a biomarker for PCP screening in at-risk patients with relevant clinical signs. Likewise, Pilkington and Sax underlined in their expert opinion that BG assay should not be used as a stand-alone test for excluding PCP in immunocompromised patients with pneumonia (Corsi-Vasquez et al., 2019).

Furthermore, the insufficient sensitivity of BG assay in non-HIV-infected patients should be considered taking into account the underlying illness. The lowest median BG level of 211 pg/ml was noticed in the HM patient subset and was significantly lower than the ones of 3,473 and 3,480 pg./mL observed in the SOT and SAIID subsets, respectively. With use of the 80 pg/ml threshold, the sensitivity and the NPV of BG assay in HM patients were then determined to be 64 and 73%, respectively. Even by changing the cutoff, the greatest sensitivity we could achieve in these patients would not exceed 82%. This finding is consistent with the results from previous studies assessing the utility of BG in other IFDs. In particular, one meta-analysis that exclusively considered HM patients revealed a sensitivity of 61% for the diagnosis of invasive aspergillosis or candidiasis (Lamoth et al., 2012). Thereafter, it has been pointed out that this suboptimal sensitivity and the subsequent low NPV were major constraints to BG use in HM patients (Giacobbe et al., 2017). Consequently, BG testing is no longer recommended to exclude aspergillosis or candidiasis in this patient population (Lamoth et al., 2021). Considering our results, this limitation could also encompass PCP.

We have also analyzed quantitative data corresponding to PCR Ct values. In accordance with the lower BG levels and consequent inferior sensitivity of BG assay described above in HM patients, we found that fungal loads were also significantly lower in this setting. In contrast, both SOT recipients and SAIID patients harbored the highest fungal loads associated with the highest BG levels. Such differences among the non-HIV-infected patient subgroups might be explained by a straight involvement of humoral and cellular immunity. Indeed, most of HM patients developing PCP have B-cell disorders, whereas SOT recipients as well as SAIID patients commonly receive chemical immunosuppressive drugs that mainly affect T-cells (Kolls, 2017; Pallet et al., 2018). It is well-known that CD4+ T-cells play a key role in the clearance of *P. jirovecii* by promoting the recruitment of phagocytes to the infected lungs (Gingerich et al., 2021). Thus, one may speculate that the high fungal loads and BG levels observed in SOT patients and SAIID patients are linked to an altered T-cell function. This hypothesis is supported by the well-known situation in HIV-infected patients in whom CD4+ T-cell level determines the PCP risk (Masur et al., 1989). The latter patients typically harbor extremely high pulmonary fungal loads associated with peak BG levels during PCP (Salzer et al., 2018; Guegan and Robert-Gangneux, 2019; Kato et al., 2019; Tasaka, 2020).

Conversely, HM patients with B-cell disorders might have preserved CD4+ T-cell activation, which could contribute to fungal clearance and therefore explain low fungal loads and BG levels in these individuals.

False negative results were reported in case of methotrexate administration (Ideguchi et al., 2020). However, this hypothesis is unlikely for our PCP cohort since none of the five patients with a negative BG result received methotrexate while the four patients who received methotrexate were BG-positive. Indeed, four out of the five negative results were observed in HM patients, underlining here again the fact that a negative BG result alone does not rule out the diagnosis of PCP in this setting. On the other hand, BG testing lacks specificity for PCP diagnosis. First, this compound is a pan-fungal biomarker and high BG levels are usually observed in IFDs other than PCP, especially invasive aspergillosis and candidiasis (Obayashi et al., 1995). Second, false positive results are commonly described, with gram-negative sepsis and IVIG treatment as main causes (Finkelman, 2020). Among the 34 PCP patients with a positive BG result, two presented with a concurrent invasive candidiasis, three had a concurrent bacterial sepsis, and one received IVIG infusions. Since BG lacks specificity for PCP, any BG-positive result requires thorough investigation of other IFDs as well as all known causes of false positive test. In other words, BG positivity should be interpreted carefully.

Several studies have attempted to correlate pulmonary fungal loads and serum BG levels in the course of PCP. Low or moderate correlations have been found either in mixed HIV-positive and HIV-negative cohorts (Desmet et al., 2009; Held et al., 2011; Engsbro et al., 2019), or in exclusively HIV-negative cohorts (de Boer et al., 2011; Szvalb et al., 2020). We found similar results with a low negative correlation coefficient of −0.37. Various hypotheses can be made to explain this discrepancy. An interval up to 10 days between BALF and serum sampling (and therefore a serum sample obtained under anti-*Pneumocystis* treatment) could be one reason for this low correlation. However, this hypothesis was deemed unlikely since the Spearman’s coefficient calculated before and after excluding patients who underwent BALF and serum sampling on the same day did not vary substantially (0.37 vs. 0.4). A more plausible explanation would be a heterogeneous release of BG from *P. jirovecii* pulmonary asci into bloodstream and/or a heterogeneous clearance of the released BG, both depending on the patient. To date, however, the exact reason why these heterogeneities do occur remains unresolved.

The main limitation of this research is the small sample size. This is due both to the single-center design of the study and the microscopic visualization of *P. jirovecii* as inclusion criteria for PCP patient group. Actually, microscopically proven PCP is a relatively uncommon event in HIV-negative patients and is related primarily to the low fungal loads in the lungs (Limper et al., 1989). However, focusing exclusively on proven PCP is essential to reinforce findings and the limitation regarding cohort size could be overcome with a multi-center scheme in the next future. Another limitation is the use of PCR Ct values and the subsequent absence of absolute quantitation to estimate the fungal burden. Since 2013, our routine PCR assay includes standards used to convert Ct values into quantitative results expressed as number of DNA copies/ml of BALF sample. However, the present study covers a 10-year period and only Ct values are available for the patients enrolled before 2013. Moreover, most of DNA extracts are no longer obtainable to any further retest. Nonetheless, in our experience, inter-series variability is low since Ct values obtained for plasmid dilutions remain in the same range whatever the run. Furthermore, as mentioned above, BG remains a pan-fungal biomarker and therefore cannot be used alone to diagnose PCP. The inclusion of a control group of HIV-negative patients presenting with IFDs other than PCP would be of interest to point out the lack of specificity of BG, which is yet admitted in clinical practice. Moreover, this was not the main objective of our study and we primarily focused our work on the lack of BG assay sensitivity for PCP diagnosis in HIV-negative patients when it is used as a stand-alone test.

It is now well recognized that BG measurement is unsatisfactory to exclude PCP diagnosis in HIV-negative individuals with consistent clinical signs (Del Corpo et al., 2020). Moreover, we have described here an even lower sensitivity of this test in patients suffering from HM. These findings point to the fact that the performance of BG assay also differs according to the disease that exposes HIV-negative individuals to PCP. Further studies should focus on these different predisposing factors. This will provide additional data to better understand BG usefulness in non-HIV-infected patients. Be that as it may, physicians should be aware that the interpretation of a negative BG result must take into account, but not be limited to, the underlying condition predisposing to PCP in non-HIV-infected patients.

## Data Availability Statement

The original contributions presented in the study are included in the article/Supplementary Material, and further inquiries can be directed to the corresponding author.

## Author Contributions

YLG and AT: conceptualization. CD, BD, and CP: data collection. CD: statistical analyses. AT: writing – original draft preparation. CD, YLG, and AT: writing – review and editing. All authors contributed to the article and approved the submitted version.

## Conflict of Interest

The authors declare that the research was conducted in the absence of any commercial or financial relationships that could be construed as a potential conflict of interest.

## Publisher’s Note

All claims expressed in this article are solely those of the authors and do not necessarily represent those of their affiliated organizations, or those of the publisher, the editors and the reviewers. Any product that may be evaluated in this article, or claim that may be made by its manufacturer, is not guaranteed or endorsed by the publisher.
