# Supplementary figures and images for: A Negative (1,3)-β-D-Glucan Result Alone Is Not Sufficient to Rule Out a Diagnosis of Pneumocystis Pneumonia in Patients With Hematological Malignancies
Source: Front Microbiol. 2021 Aug 11;12:713265. doi: 10.3389/fmicb.2021.713265 (PMC8386019; doi:10.3389/fmicb.2021.713265)

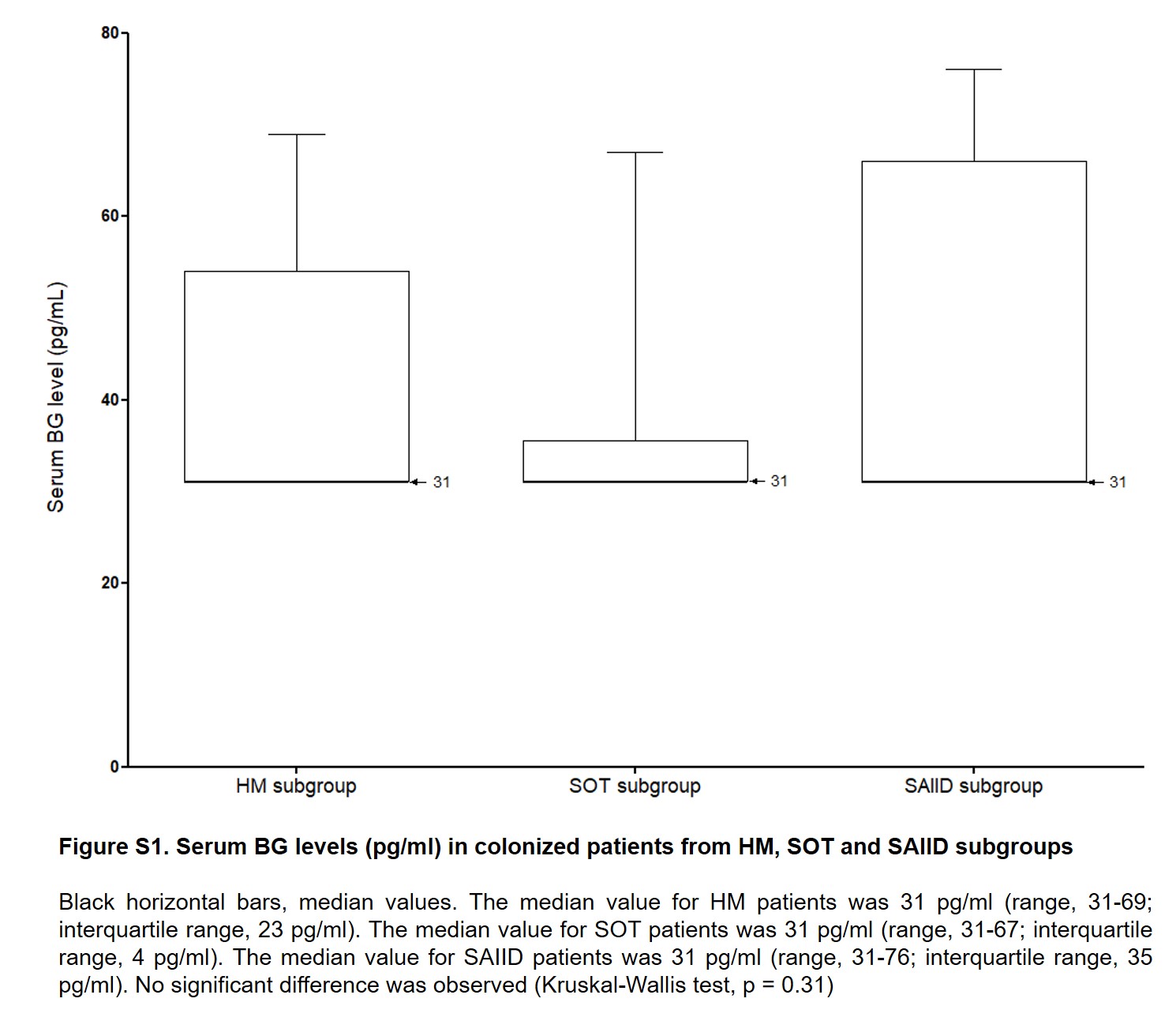

Supplement: Supplementary file 1 [file Image_1.jpg]

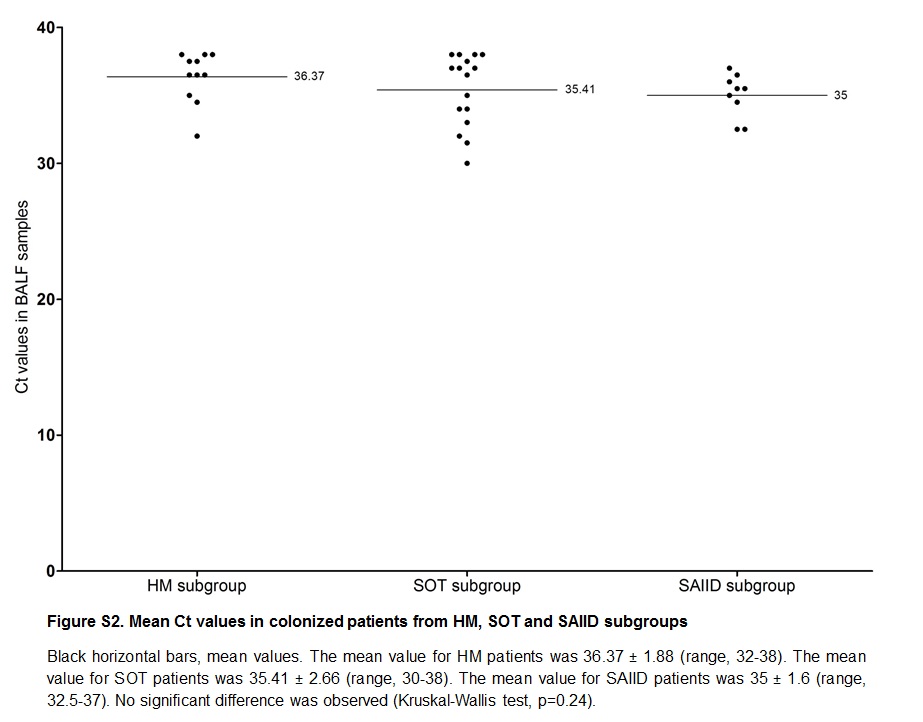

Supplement: Supplementary file 2 [file Image_2.JPEG]

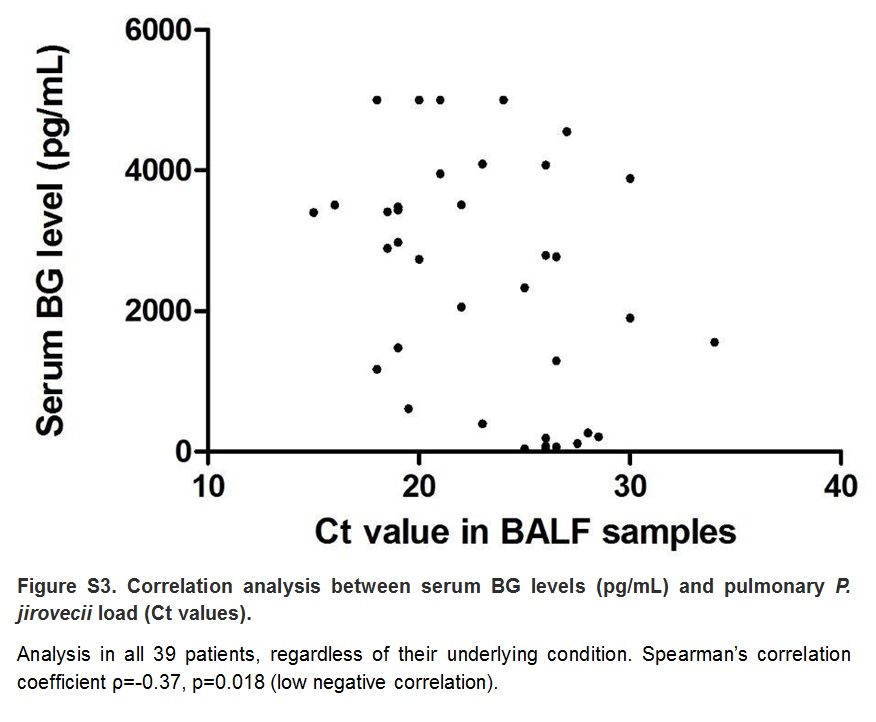

Supplement: Supplementary file 3 [file Image_3.JPEG]
